# Outer membrane protein A (OmpA) of extraintestinal pathogenic *Escherichia coli*

**DOI:** 10.1186/s13104-020-4917-5

**Published:** 2020-01-31

**Authors:** Daniel W. Nielsen, Nicole Ricker, Nicolle L. Barbieri, Heather K. Allen, Lisa K. Nolan, Catherine M. Logue

**Affiliations:** 1 Department of Veterinary Microbiology and Preventive Medicine, College of Veterinary Medicine, Iowa State University, 1802 University Blvd, Ames, IA 50011 USA; 2 Food Safety and Enteric Pathogens Research Unit, National Animal Disease Center, ARS-USDA, Ames, IA USA; 3 Department of Pathobiology, Ontario Veterinary College, University of Guelph, Guelph, ON N1G 2W1 Canada; 4 Department of Population Health, College of Veterinary Medicine, University of Georgia, 501 D.W. Brooks Drive, Athens, GA 30602 USA; 5 Department of Infectious Diseases, College of Veterinary Medicine, University of Georgia, Athens, GA 30602 USA

**Keywords:** *Escherichia coli*, Extraintestinal pathogenic *E. coli* (ExPEC), NMEC, UPEC, APEC, OmpA

## Abstract

**Objective:**

Extraintestinal Pathogenic *E. coli* (ExPEC), are responsible for host diseases such as Neonatal Meningitis *Escherichia coli* (NMEC), the second-leading cause of neonatal bacterial meningitis, Avian Pathogenic *E. coli* (APEC), a cause of extraintestinal disease in poultry, and Uropathogenic *E. coli* (UPEC), the most common cause of urinary tract infections. Virulence factors associated with NMEC include outer membrane protein A (OmpA) and type I fimbriae (FimH), which also occur in APEC and UPEC. OmpA contributes to NMEC’s ability to cross the blood–brain barrier, persist in the bloodstream and has been identified as a potential vaccine target for ExPEC, however the protein has amino acid variants, which may influence virulence of strains or alter vaccine efficacy. Although OmpA is present in virtually all *E. coli*, differences in its amino acid residues have yet to be surveyed in ExPEC.

**Results:**

Here the *ompA* gene (n = 399) from ExPEC collections were sequenced and translated in silico. Twenty-five different OmpA polymorphism patterns were identified. Seven polymorphism patterns were significantly associated with an ExPEC subpathotype, but chromosomal history most likely accounts for most differences found. The differences in OmpA protein sequences suggest that OmpA may influence variation in virulence and host specificity within ExPEC subpathotypes.

## Introduction

Members of the Extraintestinal Pathogenic *Escherichia coli* (ExPEC) pathotype are adapted for an extraintestinal lifestyle. ExPEC subpathotypes include Neonatal Meningitis *E. coli* (NMEC), Uropathogenic *E. coli* (UPEC), and Avian Pathogenic *E. coli* (APEC), which are named by the host system or species they impact [1, 2]. APEC, the causative agent of avian colibacillosis is responsible for significant morbidity, mortality, and financial losses to the poultry production worldwide [1]. UPEC is the leading cause of uncomplicated and catheter-associated urinary tract infections in humans, and serious UPEC infections can result in pyelonephritis, potentially leading to sepsis or death [3]. NMEC is the causative agent of 28–29% of neonatal bacterial meningitis cases [4, 5] with a mortality rate of 33% and survivors often suffer lifelong disability [5]. Identifying common and distinguishing virulence factors among ExPEC subpathotypes are key to explaining the pathogenesis or virulence of the pathotype or subpathotypes. One virulence factor of particular interest in ExPEC is OmpA, an outer membrane protein that promotes bloodstream survival and assists NMEC in crossing the blood brain barrier [6–8].

Structurally, OmpA consists of eight membrane-spanning β-strands that form a β-barrel [9]. The N-terminal domain consists of the first 169 amino acids and was characterized by Patutsch and Shulz [10]. The C-terminal domain was proposed to interact with the peptidoglycan layer [11], and has yet to be crystalized [12]. It has been shown that OmpA can exist as a monomer or dimer and the soluble C-terminal domain of OmpA is responsible for protein dimerization [12]. The OmpA protein forms four extracellular loops that exhibit residue patterns encoded by allelic variants in the *ompA* gene across the protein’s loops [13]. These “alleles” have been described previously [13–15]. Structurally, the OmpA loops contribute to NMEC’s survival and entry into human brain microvascular endothelial cells (HBMEC) by binding the Ecgp glycoprotein [16, 17]. Gu et al. [18] suggested that the OmpA loops might be a good vaccine target to prevent infection. OmpA also contributes to the binding and survival of NMEC in macrophages [19]. For UPEC, OmpA promotes pathogenesis associated with cystitis [20]. Additionally, OmpA contributes to binding tropism by different types of *E. coli* [21] and acts as a receptor for bacteriophages [13, 14].

Although the contribution of OmpA to NMEC pathogenesis has been demonstrated, the importance of OmpA among other ExPEC subpathotypes, such as APEC and UPEC, remains relatively underexplored. OmpA is present in virtually all *E. coli*, including commensal strains [14, 22], but is OmpA’s relationship to NMEC virulence unique and ascribable to certain polymorphisms? Are certain polymorphisms in OmpA unique to NMEC or other ExPEC? Answering such questions may provide insight into ExPEC’s ability to cause disease, its evolution, host specificity, or tissue proclivity.

This study assessed differences in OmpA amino acid sequences among ExPEC subpathotypes. An issue that might complicate such an analysis is the lack of chromosomal relatedness of the *E. coli* being compared since ExPEC subpathotypes have different phylogenetic group distributions [23]. An association of chromosomal history and polymorphism patterns in a virulence factor has precedence as polymorphisms in the adhesin FimH, a virulence factor of ExPEC, appear to correspond with phylogenetic group assignment and increased virulence [24]. Thus, this study examined OmpA amino acid sequences of ExPEC assigned using Clermont’s 2013 analysis.

## Main text

### Materials and methods

#### ExPEC strains and DNA isolation

A total of 399 ExPEC were used in this study randomly selected from APEC, NMEC, and UPEC collections previously described [25–28]. All isolates were phylogenetically grouped by Clermont’s phylogenetic typing scheme (Additional file 1: Table S1) [23, 25]. DNA template was prepared as described previously [25].

#### *ompA* gene amplification and sequencing

The *ompA* gene was amplified from each strain twice via PCR with two primer sets and PCR reactions (Additional file 2: Table S2). PCR conditions were 94 °C for 3 min, followed by 30 cycles of amplification (denaturation: 30 s at 94 °C, annealing: 30 s at 54 °C, extension: 72 °C for 90 s), and a final extension at 72 °C for 7 min using a MasterCycler Gradient thermocycler (Eppendorf, Germany). 10 µl of PCR products were confirmed on a 2% agarose gel in 1x TAE buffer and remaining PCR products purified using ExoSAP-IT (Affymetrix, ThermoFisher) to remove primers and dNTPs before they were Sanger sequenced at the Iowa State University DNA Sequencing Facility (Ames, IA).

#### In silico analysis of *ompA*

Nucleotide sequences of *ompA* were imported into Geneious (v. 10.2, BioMatters LTD, Auckland, New Zealand) aligned, trimmed for consistent length and translated in silico. Residues were aligned using the Geneious aligner with the Blosum 62 cost matrix, and non-unique residues removed. Polymorphisms at any position occurring fewer than three times among all OmpA sequences were interpreted as potential sequencing errors and excluded from analysis. The resulting amino acid sequences were used as polymorphism pattern strings and imported into R for analysis. TidyVerse and ggplot2 packages were used to conduct analyses and generate figures [29, 30]. Data regarding isolate, subpathotype, polymorphism patterns, and phylogenetic group assignment is contained in Additional file 3.

#### Statistical analysis

The Chi square test of homogeneity was used to determine statistically significant differences among the ExPEC subpathotypes for any polymorphism pattern which occurred greater than 20 times. Significance for all statistical tests was determined at the α = 0.05 level.

### Results and discussion

#### The OmpA protein has unique polymorphism patterns

**Fig. 1:**
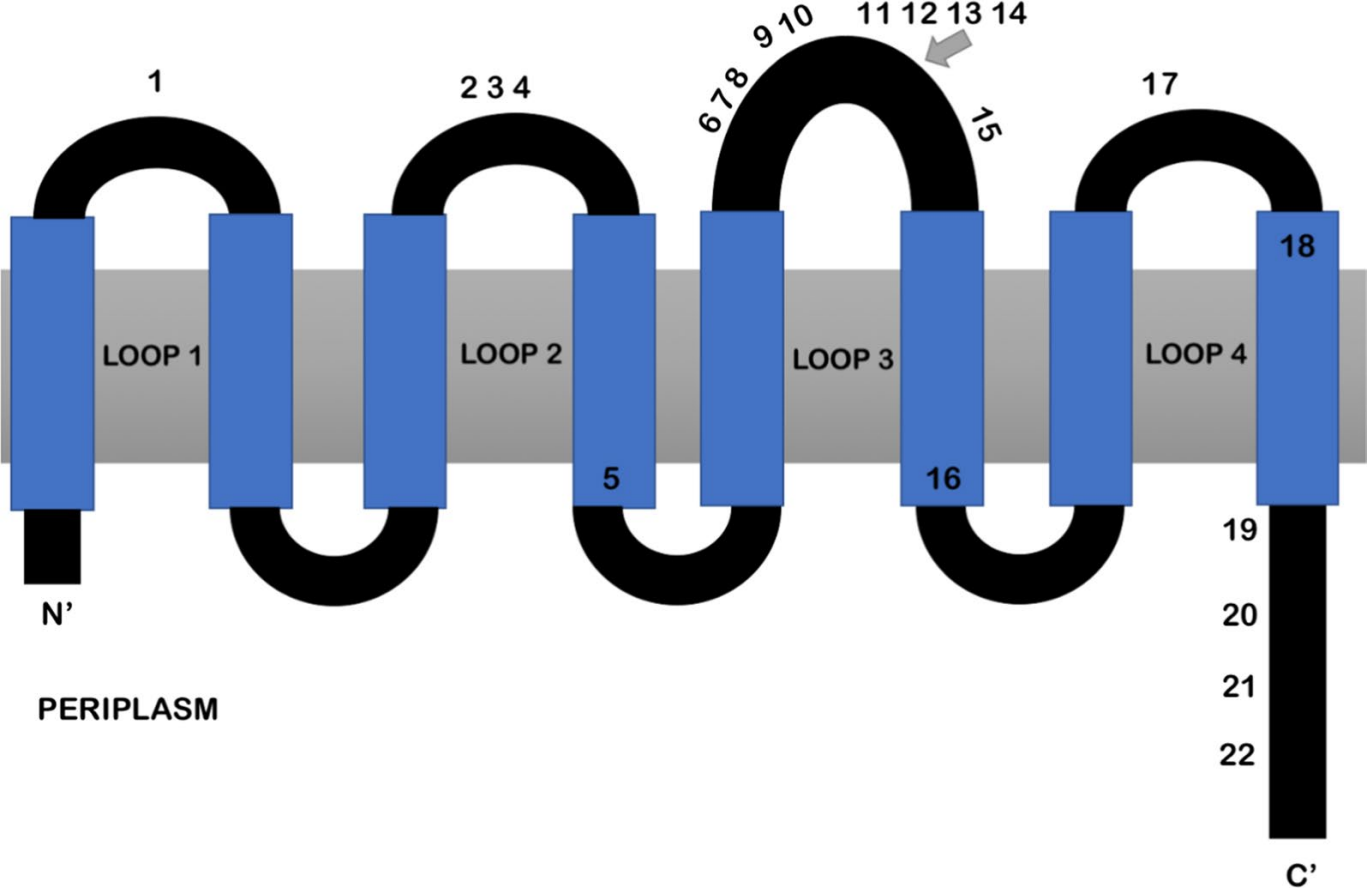
Structure of OmpA, represented by the black and blue line looping through the outer membrane, with amino acid sequence polymorphisms indicated at their approximate positions. Polymorphisms 1–18 are within the N terminal domain region while polymorphisms 19–22 are within the linker/dimerization domain. The OmpA structure is based on data presented in other work [10, 21]

Analysis of the *ompA* sequences identified 22 different OmpA predicted polymorphism sites among all ExPEC strains examined (Fig. 1). Most OmpA polymorphisms were located within the N-terminus region or the loops of the protein, which have previously been designated as part of the N-terminal domain (Fig. 1). Polymorphism patterns were identified based on the unique string of polymorphisms for each isolate, and each polymorphism pattern was assigned an N-terminus (identified by letters) and dimerization region (identified by numbers) as previously characterized in the literature (Additional file 4: Table S3) [13–15, 21]. OmpA patterns were compared to *Escherichia coli*, MG1655 (Genbank: U00096.3) [31, 32] and 9% of ExPEC had the same polymorphism pattern (E2) (Additional file 4: Table S3). The OmpA sequence is: MKKTAIAIAVALAGFATVAQAAPKDNTWYTGAKLGWSQYHDTGFINNNGPTHENQLGAGAFGGYQVNPYVGFEMGYDWLGRMPYKGSVENGAYKAQGVQLTAKLGYPITDDLDIYTRLGGMVWRADTKSNVYGKNHDTGVSPVFAGGVEYAITPEIATRLEYQWTNNIGDAHTIGTRPDNGMLSLGVSYRFGQGEAAPVVAPAPAPAPEVQTKHFTLKSDVLFNFNKATLKPEGQAALDQLYSQLSNLDPKDGSVVVLGYTDRIGSDAYNQGLSERRAQSVVDYLISKGIPADKISARGMGESNPVTGNTCDNVKQRAALIDCLAPDRRVEIEVKGIKD. All polymorphisms identified in this study except for the polymorphism numbered 21 were previously described in the literature [21]. Polymorphism pattern B5 encoded a valine at this position, while all other polymorphism patterns encoded an alanine. This pattern was found in 3.5% of the APEC but was absent in NMEC and UPEC strains (Additional file 4: Table S3).

#### Polymorphism patterns can vary with the ExPEC subpathotype

**Fig. 2:**
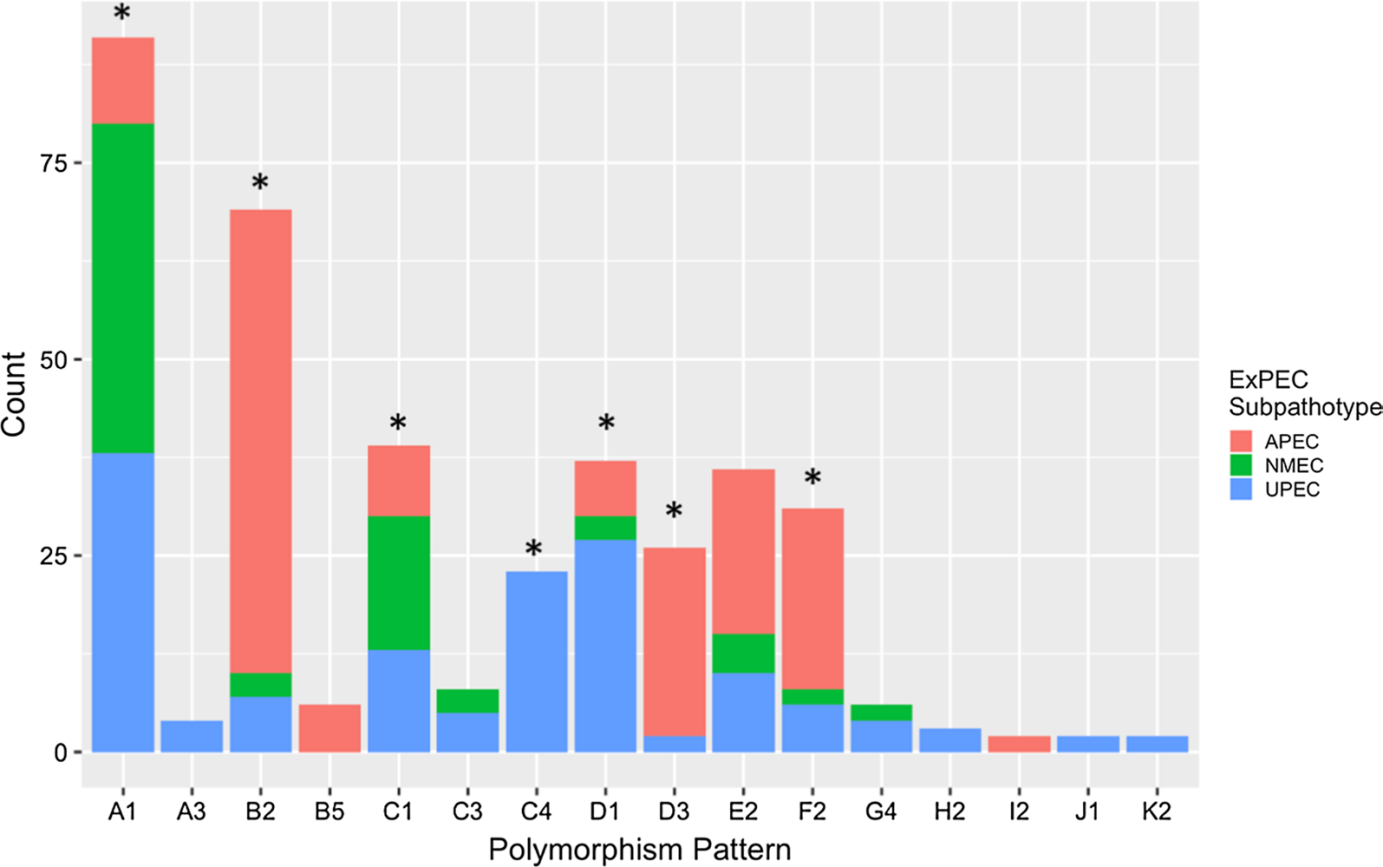
Polymorphism patterns and prevalence of each pattern for APEC (n = 171), NMEC (n = 80), and UPEC (n = 148) for any polymorphism pattern that occurred greater than once. Polymorphism patterns A1, B2, C1, C4, D1, D3, and F2 are statistically significant between the subpathotypes (p < 0.05). Any polymorphism pattern that occurred fewer than two times was excluded from analysis

Statistically significant differences were observed in the distribution of seven polymorphism patterns among APEC, NMEC, and UPEC examined (Fig. 2). APEC were more likely to exhibit OmpA polymorphism patterns B2, D3, E2, and F2; whereas, UPEC were likely to exhibit patterns A1, A3, C4, D1, G4, and H2. The majority of NMEC contained OmpA polymorphism pattern A1, but NMEC also had a greater relative prevalence of polymorphism patterns B2, C1, and C3 than one or more of the other subpathotypes (Fig. 2). Although most of these differences were statistically significant, the composition of the phylogenetic groups within the ExPEC subpathotypes differed [25] and as a result, polymorphism patterns of APEC, NMEC, and UPEC were analyzed against phylogenetic group assignment.

#### Polymorphism patterns are associated with ExPEC of different subpathotypes and phylogenetic groups provide additional resolution

**Fig. 3:**
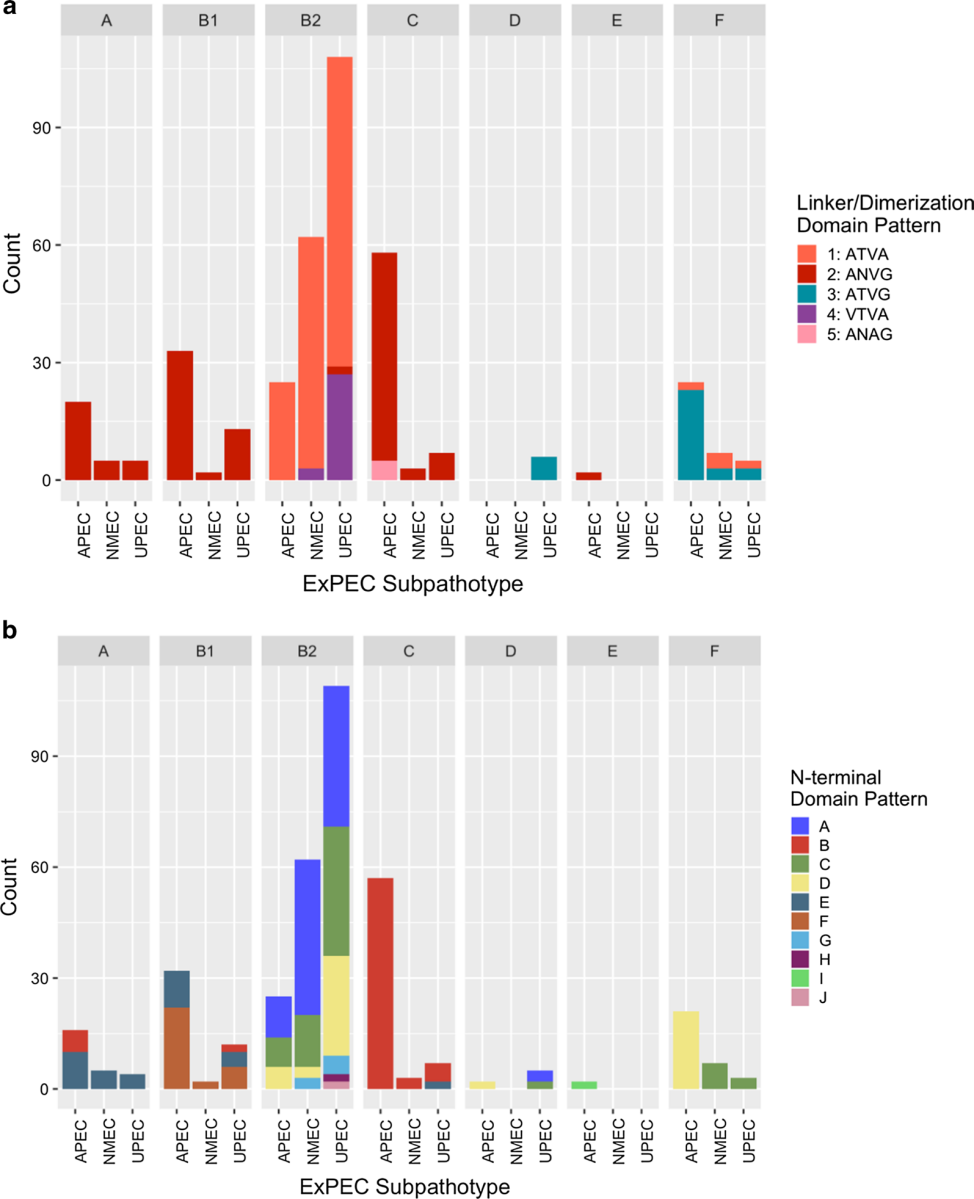
ExPEC subpathotype polymorphisms differ across their phylogenetic groups (facetted plots) by their linker/dimerization (**a**) and N-terminal domains (**b**). Any polymorphism pattern that occurred fewer than two times per subpathotype was excluded from analysis

The OmpA protein sequences identified in our ExPEC collection could sometimes predict the phylogenetic group assignment (Fig. 3 and Additional file 5: Figure S1). When the linker/dimerization domains were examined for relationship to phylogenetic group assignment, distinctions were observed among the subpathotypes (Fig. 3a). Phylogenetic groups A and B1 were unanimously composed of the ANVG linker/dimerization polymorphism pattern. The dimerization pattern for phylogenetic group C included an additional unique dimerization pattern, ANAG, and this pattern was only found in APEC (Fig. 3a). There were also differences in the linker/dimerization domains of phylogenetic group B2 as NMEC and UPEC contained the unique polymorphism pattern VTVA, which was absent from APEC. However, the proportion of NMEC and UPEC assigned to phylogenetic group B2 is greater than that of APEC (Additional file 1: Table S1) as noted previously [25, 27]. Phylogenetic group F consisted of ATVA and ATVG. A majority of APEC belong to phylogenetic group C [25], so it was unsurprising to find APEC had a second polymorphism pattern compared to NMEC and UPEC, identified by the two linker/dimerization domain patterns ANAG and ANVG (Fig. 3a).

When the N-terminal domain pattern was examined, differences between the ExPEC subpathotypes were evident for some of the phylogenetic groups (Fig. 3b). Important subpathotype differences in OmpA polymorphisms were found in APEC assigned to phylogenetic group A, and these APEC had the “B” pattern for their N-terminal domain unlike NMEC and UPEC, but UPEC also had a “B” N-terminus pattern unlike APEC and NMEC. ExPEC subpathotypes assigned to the B2 and F phylogenetic groups also had differences. The UPEC phylogenetic group B2 had a greater diversity of polymorphism patterns, and APEC had a different N-terminal domain pattern in phylogenetic group F compared to NMEC and UPEC. This pattern, N-terminal domain pattern “D” was shared with phylogenetic group B2 of APEC and NMEC isolates as well as phylogenetic group D isolates of APEC. Therefore, a subset of APEC of different chromosomal lineages harbor OmpA proteins, similar to those of NMEC and UPEC in the B2 phylogenetic group. Alternatively, the phylogenetic classification scheme assigning isolates may have insufficient resolution for some of the strains surveyed.

The OmpA loops of NMEC have been shown to contribute to neonatal bacterial meningitis [16, 33]. Mittal et al. [33] found that loops 1 and 3 were necessary for survival in macrophages; loops 1 and 2 were necessary for meningitis, and alterations of loop 4 resulted in enhanced severity in NMEC’s pathogenesis. Nevertheless, this study found no defining loop pattern for NMEC, suggesting that an NMEC OmpA-targeting vaccine may not be widely efficacious [18]. Like NMEC, the APEC and UPEC subpathotypes did not have one defining polymorphism pattern for the subpathotype. There were, however, statistically significant differences between some polymorphism patterns and their ExPEC subpathotypes, which agrees with the assessment that certain subpathotype subsets can be eliminated as zoonotic pathogens (Fig. 2) [28]. The lack of any subpathotype-only OmpA types also provides further evidence of a zoonotic potential of these organisms [34–37].

Although the different ExPEC subpathotypes did have significantly different OmpA polymorphism patterns, these patterns were often associated with the phylogenetic groups. However, there were differences found between avian and human ExPEC for some phylogenetic group isolates. APEC belonging to phylogenetic group F had an N-terminus pattern unlike NMEC and UPEC (Fig. 3). For isolates belonging to phylogenetic group C, UPEC had a unique N-terminus pattern, and APEC had a unique linker/dimerization domain. Although the unique differences observed cannot be accounted, they may have potential to confer environmental or pathogenic advantage to strains possessing them, warranting further investigation. As the phylogenetic groups were unable to sufficiently define all OmpA patterns, this study suggests there may be selective pressures on the protein or that the creation of a new phylogenetic group is warranted.

In conclusion, this study identified 22 polymorphisms and 25 polymorphism patterns among APEC, NMEC, and UPEC subpathotypes. APEC, NMEC, and UPEC did not have specific conserved OmpA polymorphism patterns, but some were found solely within a subpathotype and certain OmpA polymorphism patterns were associated with certain phylogenetic groups. For NMEC, there was no conserved OmpA polymorphism pattern, prompting questions regarding OmpA’s role in crossing the blood brain barrier and survival. Further work is needed to demonstrate the biological significance of OmpA polymorphisms, but this study provides an important first step in elucidating the relationships between amino acid differences and their respective function.

## Limitations

This study is based on analysis of a collection of NMEC, APEC and UPEC randomly selected from collections described previously. The data can be viewed as being slightly biased based on the strain types examined—not all of the Clermont phylogenetic groups are represented in a subpathotype reflecting the majority of strains causing disease in a host. The study provides insight into OmpA as virulence factor of ExPEC, polymorphism patterns and their association with subpathotypes and phylogenetic group classification.

## Supplementary information

**Additional file 1: Table S1.** Strain information for the ExPEC examined.

**Additional file 2: Table S2**. PCR primers and reagents.

**Additional file 3.** Sequences for OmpA analyzed including the strain ID, ompA sequence, non dimer and dimer regions, polymorphisms identified, sub pathotype of the strains, polymorphism pattern ID, and phylogenetic group.

**Additional file 4: Table S3.** Polymorphism pattern identifier with the polymorphism pattern string and the number of times the pattern occurred within the ExPEC examined.

**Additional file 5: Figure S1.** Polymorphism patterns for the ExPEC subpathotypes separated by phylogenetic group. The same polymorphism pattern often occurred within the same phylogenetic group. Any polymorphism pattern that occurred fewer than two times per subpathotype was excluded from analysis.

## Data Availability

The data sets used and/or analyzed for this study are available from the corresponding author on reasonable request.

## Abbreviations

APEC: Avian Pathogenic *Escherichia coli*
NMEC: Neonatal meningitis *Escherichia coli*
UPEC: Uropathogenic *Escherichia coli*
ExPEC: Extraintestinal Pathogenic *Escherichia coli*
OmpA: Outer membrane protein A
