# Supplementary material for: Outer membrane protein A (OmpA) of extraintestinal pathogenic Escherichia coli
Source: BMC Res Notes. 2020 Jan 31;13:51. doi: 10.1186/s13104-020-4917-5 (PMC6995065; doi:10.1186/s13104-020-4917-5)
Supplement: Supplementary file 1 — Additional file 1: Table S1. Strain information for the ExPEC examined. [file 13104_2020_4917_MOESM1_ESM.docx]

Table S1: Strain information for the ExPEC examined.

|  | **Phylogenetic Assignment** | | | | | | | |  |
| --- | --- | --- | --- | --- | --- | --- | --- | --- | --- |
|  | **A** | **B1** | **B2** | **C** | **D** | **E** | **F** | **Unknown** | **Total** |
| **APEC** | 21 | 33 | 26 | 59 | 3 | 2 | 25 | 2 | 171 |
| **NMEC** | 5 | 2 | 62 | 3 | 0 | 1 | 7 | 0 | 80 |
| **UPEC** | 5 | 13 | 109 | 7 | 7 | 1 | 6 | 0 | 148 |
| **ExPEC Total** | 31 | 48 | 197 | 69 | 10 | 4 | 38 | 2 | 399 |
| **Prevalence** | 7.8% | 12.0% | 49.4% | 17.3% | 2.5% | 1.0% | 9.5% | 0.5% | 100% |
