# Supplementary material for: Outer membrane protein A (OmpA) of extraintestinal pathogenic Escherichia coli
Source: BMC Res Notes. 2020 Jan 31;13:51. doi: 10.1186/s13104-020-4917-5 (PMC6995065; doi:10.1186/s13104-020-4917-5)
Supplement: Supplementary file 2 — Additional file 2: Table S2. PCR primers and reagents. [file 13104_2020_4917_MOESM2_ESM.docx]

Table S2: PCR Primers and Reagents

| Primer Set | Polymerase Used | Source |
| --- | --- | --- |
| GTTATCTCGTTGGAGATATTCATGG  GCGGGGTTTTTCTACCAGAC | USBtaq (Affymetrix) | This Study |
| CACTGGCTGGTTTCGCTAC  GCGGCTGAGTTACAACGTCT | DreamTaq (Thermo Scientific) | Liao *et al*. 2017 |
