# Supplementary material for: Outer membrane protein A (OmpA) of extraintestinal pathogenic Escherichia coli
Source: BMC Res Notes. 2020 Jan 31;13:51. doi: 10.1186/s13104-020-4917-5 (PMC6995065; doi:10.1186/s13104-020-4917-5)
Supplement: Supplementary file 4 — Additional file 4: Table S3. Polymorphism pattern identifier with the polymorphism pattern string and the number of times the pattern occurred within the ExPEC examined. [file 13104_2020_4917_MOESM4_ESM.docx]

Table S3: Polymorphism pattern identifier with the polymorphism pattern string and the number of times the pattern occurred within the ExPEC examined.

| **Polymorphism Pattern** | **Sequence** | **Total**  (n=399) | **APEC** (n=171) | **NMEC** (n=80) | **UPEC**  (n=148) |
| --- | --- | --- | --- | --- | --- |
| A1 | NSVEVS--VY----NYHMATVA | 91 | 11 | 42 | 38 |
| A3 | NSVEVS--VY----NYHMATVG | 4 | 0 | 0 | 4 |
| B2 | PDNIVA--VPGASFDYHMANVG | 69 | 59 | 3 | 7 |
| B5 | PDNIVA--VPGASFDYHMANAG | 6 | 6 | 0 | 0 |
| C1 | PDNIIA--VPGASFDYHMATVA | 39 | 9 | 17 | 13 |
| C3 | PDNIIA--VPGASFDYHMATVG | 8 | 0 | 3 | 5 |
| C4 | PDNIIA--VPGASFDYHMVTVA | 24 | 0 | 1 | 23 |
| D1 | NSVEVS--FD----NYHMATVA | 37 | 7 | 3 | 27 |
| D3 | NSVEVS--FD----NYHMATVG | 26 | 24 | 0 | 2 |
| E2 | NSVEIS--VY----NYHMANVG | 36 | 21 | 5 | 10 |
| F2 | PDNIVA--VPGASFDYNLANVG | 31 | 23 | 2 | 6 |
| G1 | DSVEVS--VY----NYHMATVA | 2 | 0 | 1 | 1 |
| G4 | DSVEVS--VY----NYHMVTVA | 7 | 1 | 2 | 4 |
| H2 | PDNIIA--VPGASFDYNLANVG | 4 | 1 | 0 | 3 |
| I2 | DSVEVAHNVT-ESENWHLANVG | 3 | 2 | 1 | 0 |
| J1 | DSVEVS--FD----NYHMATVA | 2 | 0 | 0 | 2 |
| J3 | DSVEVS--FD----NYHMATVG | 1 | 1 | 0 | 0 |
| K2 | DDNIIA--VPGASFDYHLANVG | 2 | 0 | 0 | 2 |
| L2 | NDNIVA--VPGASFDYNLANVG | 1 | 1 | 0 | 0 |
| M1 | NDNIVS--VY----NYHMATVA | 1 | 1 | 0 | 0 |
| N2 | NSVAIS--VY----NYHMANVG | 1 | 1 | 0 | 0 |
| O3 | NSVKVS--FD----NYHMATVG | 1 | 1 | 0 | 0 |
| P2 | NSVQIS--VY----NYHMANVG | 1 | 1 | 0 | 0 |
| Q2 | PDDIVA--VPGASFDYHMANVG | 1 | 0 | 0 | 1 |
| R2 | PDNIVA--VPGASFDYHLANVG | 1 | 1 | 0 | 0 |
