# Supplementary material for: Outer membrane protein A (OmpA) of extraintestinal pathogenic Escherichia coli
Source: BMC Res Notes. 2020 Jan 31;13:51. doi: 10.1186/s13104-020-4917-5 (PMC6995065; doi:10.1186/s13104-020-4917-5)
Supplement: Supplementary file 5 — Additional file 5: Figure S1. Polymorphism patterns for the ExPEC subpathotypes separated by phylogenetic group. The same polymorphism pattern often occurred within the same phylogenetic group. Any polymorphism pattern that occurred fewer than two times per subpathotype was excluded from analysis. [file 13104_2020_4917_MOESM5_ESM.docx]

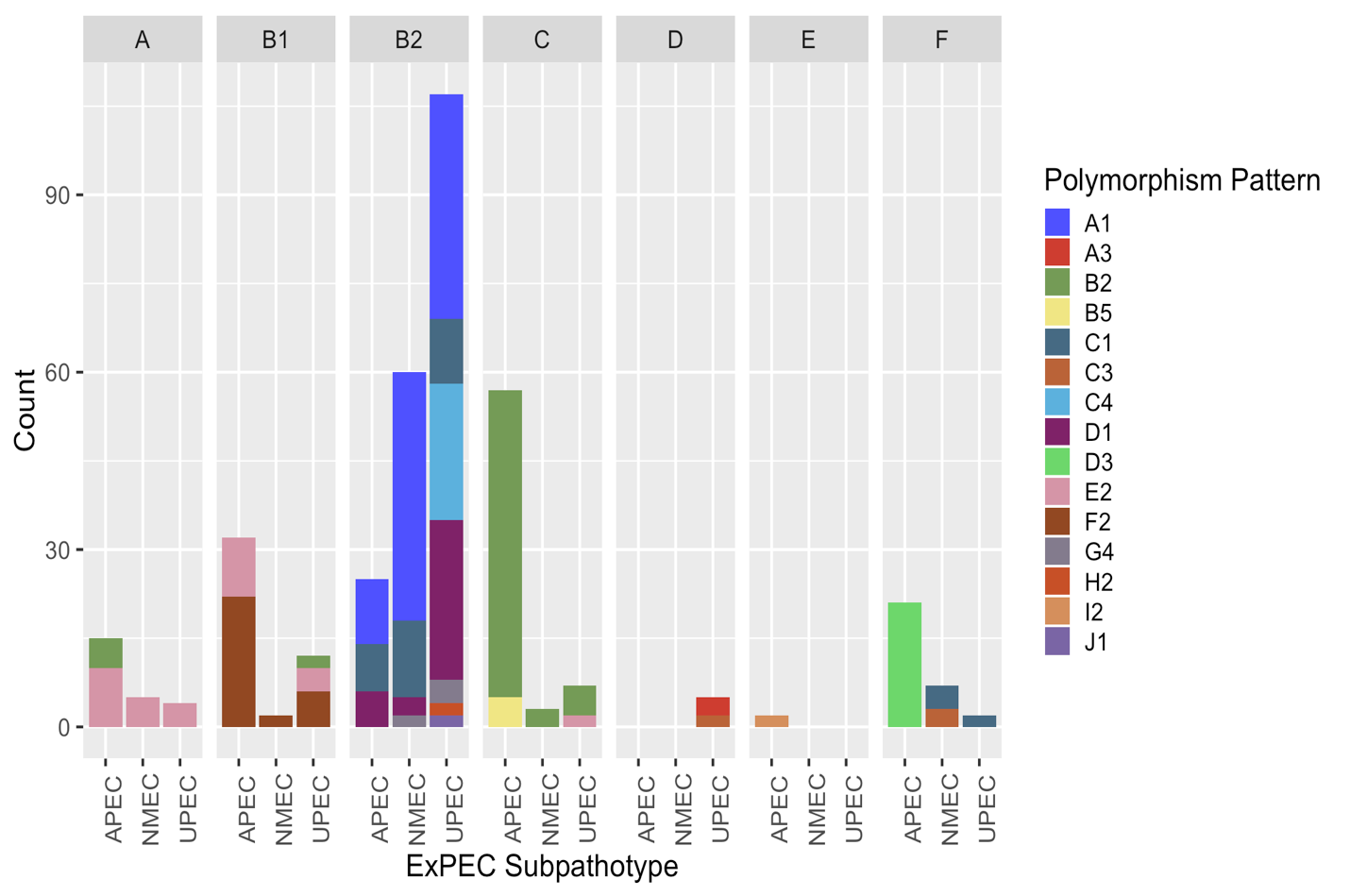


Figure S1: Polymorphism patterns for the ExPEC subpathotypes separated by phylogenetic group. The same polymorphism pattern often occurred within the same phylogenetic group. Any polymorphism pattern that occurred fewer than two times per subpathotype was excluded from analysis.
